# Exploring the key genes of colorectal “adenoma-cancer” based on graph transformer

**DOI:** 10.1371/journal.pone.0359785

**Published:** 2026-10-05

**Authors:** Yue Yuan, Yu Liu, Jingchun Fan, Zhen Ren

**Affiliations:** 1 School of Information Engineering, Gansu University of Chinese Medicine, Lanzhou, China; 2 Center for Evidence-based Medicine, Gansu University of Chinese Medicine, Lanzhou, China; Huazhong Agriculture University, CHINA

## Abstract

Colorectal cancer (CRC) is one of the most lethal malignancies worldwide, and the precise identification of biomarkers from colonic adenoma to cancer is of great significance for preventing the development of adenocarcinoma. Given that existing methods inadequately capture the topological network relationships among genes, this study proposes a graph neural network model based on multifeature learning, named ChebTs, to investigate the correlation between key genes involved in the colorectal “adenoma-cancer” transition. The GSE41657 and GSE31905 datasets from the GEO database were stratified into normal, adenoma, and colorectal cancer groups. Feature encoding was introduced to enhance node features, followed by Gene Ontology (GO) and Kyoto Encyclopedia of Genes and Genomes (KEGG) enrichment analyses of differentially expressed genes (DEGs). A protein-protein interaction (PPI) network was constructed using Cytoscape software, and the aggregated information was embedded into the model for training to generate a list of key genes with corresponding importance scores. An attention pooling mechanism aggregated node-level representations into graph level representations for sample classification, and a two layer fully connected network, following activation and regularization, produced predicted probabilities. Furthermore, we provided interpretable analyses at the gene level using GNNExplainer. The results were validated through virtual knockout techniques. The eight screened genes, Fibronectin 1 (*FN1*), Claudin 2 (*CLDN2*), Interleukin-33 (*IL-33*), Matrix Metallopeptidase 1 (*MMP1*), Stanniocalcin 2 (*STC2*), Insulin-Like Growth Factor-Binding Protein 7 (*IGFBP7*), NADPH Oxidase 4 (*NOX4*), and Secreted Frizzled Related Protein 1 (*SFRP1*), were all found to be associated with overall survival (OS) in CRC. In this study, eight molecules closely related to the development of colorectal adenocarcinoma were screened out, and they may be diagnostic biomarkers of colorectal cancer. These genes affect the prognosis of patients by participating in biological processes such as remodeling of extracellular mechanisms, and are of great significance for preventing the carcinogenesis of adenoma.

## Introduction

Colorectal cancer (CRC) is one of the most prevalent and lethal malignancies worldwide, and its pathogenesis is characterized by a distinct stepwise progression, with the “adenoma-cancer” sequence being recognized as the classical paradigm colorectal tumor development [1]. The mutational process by which normal mucosa evolves into invasive carcinoma through adenomatous polyps is driven by a series of genomic alterations affecting cancer related pathways. However, this transformation process is also influenced by the gut microbiota. Shi et al. [2] discovered that diosgenin oligosaccharides from Dendrobium officinale could alleviate chronic colitis by regulating the inflammatory pathways and gut microbiota. Establishing gene regulatory networks through graph structures can better understand the above complex biological theories. Therefore, the precise identification of key genes involved in this process is essential not only for elucidating the molecular mechanisms underlying colorectal carcinogenesis but also for providing a theoretical basis for the discovery of early diagnostic biomarkers and the implementation of personalized therapeutic strategies.

In recent years, the identification of key genes has relied primarily on two major approaches. The first approach is based on differential expression analysis and mutation spectrum profiling using high throughput sequencing data. By comparing omics data from normal mucosa, adenomas, and carcinomas, this strategy enables the screening of genes that exhibit sustained differential expression or mutation enrichment throughout the sequential transformation process. The second approach relies on functional validation in animal models, where gene editing techniques are employed to simulate specific mutation combinations and to observe their effects on malignant transformation of cells [3]. However, existing methods have certain limitations in identifying core genes. Traditional differential expression analysis fails to account for the complex network structures formed by gene-gene interactions, co-expression, or pathway associations. Tumor development, in essence, reflects an imbalance in network regulatory systems. Furthermore, high throughput omics data are characterized by high dimensionality, small sample sizes, high noise levels, and sparsity. Machine learning based screening methods applied to such data are prone to issues such as the “curse of dimensionality”, leading to model overfitting and poor generalization ability [4].

Graph Neural Networks (GNNs) have demonstrated considerable efficacy in processing graph structured data with non-Euclidean geometries. Their core principle involves learning low dimensional vector representations of target nodes through iterative aggregation of information from neighboring nodes. Through a multi-layer message passing mechanism, they capture topological dependencies among genes and incorporate network based prior knowledge into the learning process, thereby achieving information enhancement and noise reduction in high dimensional, sparse expression data [5]. EMOGI [6] is an interpretable model based on graph convolutional neural networks (GCNs) that classifies cancer driving genes by integrating features such as DNA methylation data, gene expression data, and PPI networks. However, its use of a single variant of GNN prevents it from capturing global information. MTGCN [7] introduces a ChebNet based graph convolutional network that identifies cancer driver genes through joint optimization of node classification and link prediction tasks, yet its lack of structural encoding limits its capacity to capture gene-gene dependencies. DGMP [8] combines directed graphs and MLPs to learn node features from gene regulatory networks and omics data for key gene prediction. However, its use of global average pooling results in the loss of structural information. To address these limitations, this study proposes a new GNN model, ChebNetII, based on Chebyshev interpolation and combined with a Transformer to identify key genes in colorectal “adenoma-cancer” progression. By embedding various types of encodings to enrich node information and integrating ChebNetII with the Transformer, this approach offers significant advantages in capturing long range dependencies among genes and perceiving the topological structure of the graph, thereby providing a new perspective for investigating biomarkers that drive the adenoma to cancer transformation process.

## Materials and methods

### Data collection

The microarray datasets GSE41657 and GSE31905 used in the present study were sourced from the GEO database [9]. The original CEL files were converted into expression matrices at the probe level using the GEOquery package. Both datasets were generated using the GPL6480 platform (Agilent-014850 Whole Human Genome Microarray 4x44K G4112F), and a total of 155 samples were included after dataset merging. Specifically, GSE41657 consists of human colorectal tissue specimens obtained by colonoscopic resection, comprising 51 adenomas, 25 colorectal cancer samples, and 12 normal mucosa samples. GSE31905 was established to identify prognostic gene expression biomarkers for early stage primary colorectal cancer and provides expression profiles for 55 colorectal cancer samples, 7 normal samples, and 5 adenomas.

### Data processing

#### DEGs analysis.

Data preprocessing was performed using the sva and ComBat packages in R Studio (version 4.5.1), encompassing data cleaning, normalization, log transformation, and batch effect correction. When the probes were mapped to gene symbols, no obvious missing values were found. The merged dataset was corrected for batch effects using principal component analysis, to ensure high quality data for subsequent model construction. Differential expression analysis of the datasets was conducted using the limma and ggplot2 packages, in conjunction with the GEO2R tool. Three pairwise comparisons of DEGs were established, normal vs adenoma, adenoma vs carcinoma, and normal vs carcinoma. To ensure robustness of the results, the screening criteria were set at $\left|\log_{2}FC\right|>1$ and *P* < 0.05, which were considered statistically significant.

#### Construction of the PPI network.

Differentially expressed genes were extracted using the rbioapr and dplyr packages in R Studio. PPI network data were obtained from the STRING database (version 12.0) [10], and only interactions with a combined score greater than 0.4 were retained [9]. The resulting network data were then imported into Cytoscape for visualization.

### Overview of the model

We propose a graph neural network model, named ChebTs, that combines local topological encoding with a global attention mechanism to identify key genes in colorectal adenoma to cancer progression. Among them, ChebTs consists of ChebNetII, Transformer layers and attention pooling layers. Enhanced gene features were obtained by aggregating original expression data (node features and PPI network) with Laplacian positional encodings (PE) and random walk structural encodings (SE), allowing the model to learn from both expression profiles and network topology. The three inputs were independently projected into a unified space via linear projection layers, and dropout was applied to generate initial node representations (dropout = 0.3).

To identify key genes, the ChebNetII convolutional layer (K = 5) was used to aggregate local neighborhood information. A normalized adjacency matrix was constructed using PPI edge indices and the maximum eigenvalue of the Laplacian matrix, and the Chebyshev polynomial recursion was subsequently applied. Following SiLU activation and residual connections, the updated node representations were fed into a Transformer layer (L = 2) with 4 head attention per layer, ultimately generating a list of key genes with corresponding importance scores. An attention pooling mechanism based on these gene importance scores was then used to aggregate node level representations into graph level representations for sample classification. Finally, a two layer fully connected network (MLP), following activation and regularization, produced predicted probabilities from the graph level representations. The overall model architecture is illustrated in Fig 1.

**Fig 1:**
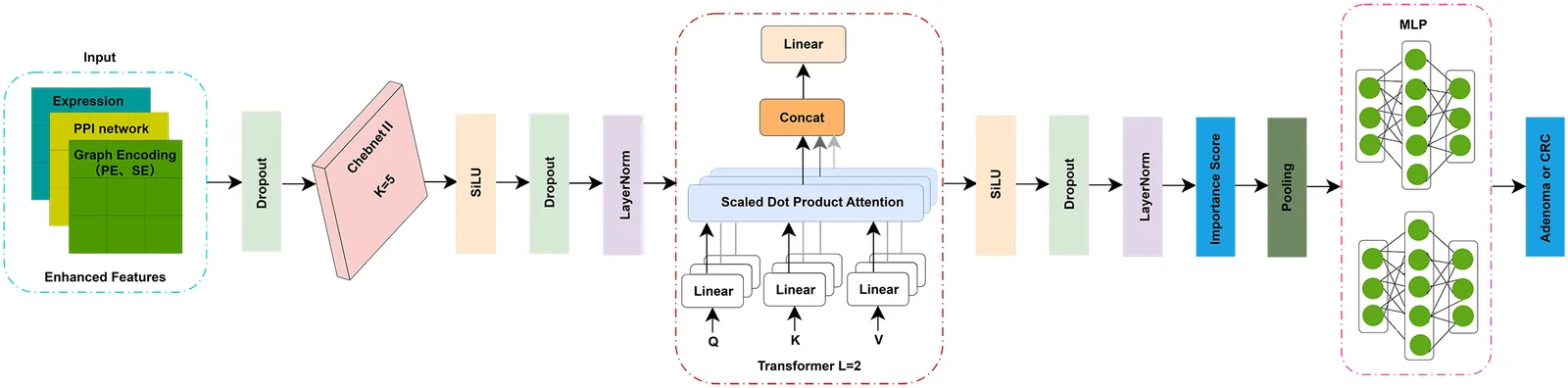
Model architecture diagram. In this study, different genetic data were combined into an undirected network graph. After introducing Laplacian feature encoding, an enhanced feature set was formed for ChebTs input, and a list of key genes was obtained. Finally, a multi-layer perceptron classified the graph structure based on the gene importance scores.

#### Experimental environment and hyperparameter.

All experiments in this study were conducted on an NVIDIA GeForce GTX 4080 GPU. The proposed model was implemented using the PyTorch framework, and a 5 fold cross validation strategy was adopted, with the dataset partitioned into training, validation, and test sets at a ratio of 6:2:2. The AdamW optimizer was employed with a learning rate of $3\times 10^{-4}$, hidden dimension of 128, and the model was trained for 200 epochs. To enhance model robustness and generalization performance, a learning rate scheduler incorporating warmup and cosine annealing strategies was applied. Additionally, gradient clipping was implemented to prevent gradient explosion resulting from message passing in the deep graph neural network architecture.

#### Coding of features.

Positional encodings are designed to provide information about the position of a given node within the graph. In this study, the normalized Laplacian matrix of the graph was computed to capture hierarchical positional information for each node, we define normalized magnetic Laplacian as formula Eq (1). The positional encoding matrix is defined as $𝐏\in {\mathbb{R}}^{N\times k}$. Structural encodings, in contrast, are intended to provide graph structure embeddings by enabling the transfer of richer information to each node through random walk based structural encodings, thereby enhancing the generalization capacity of the graph neural network. Consequently, when two nodes share similar subgraphs, their respective PE and SE are expected to be closely aligned [11].


$$\begin{array}{c} L=I-D^{-\frac{1}{2}}AD^{-\frac{1}{2}} \end{array}$$ (1)

Where *I* denotes the identity matrix, *D* represents the degree matrix, and *A* is the adjacency matrix.

#### ChebNetII model.

GNNs have become one of the most powerful tools for a wide range of graph based learning tasks due to their ability to aggregate local information through message passing mechanisms. However, the illegal coefficients learned by ChebNet when approximating analytical filter functions can easily lead to overfitting. At the same time, most methods still rely on semi-supervised learning, and the scarcity of labels limits their applicability in real world systems. Although GCN can expand the receptive field through multiple layers of stacking, each layer of GCN can only aggregate 1-hop neighborhood information, thus unable to learn the discriminative information of high-pass filtering representation. The attention heads in GAT perform attention-driven feature aggregation on the graph structure based on node features and learnable parameters. Therefore, its spectral characteristics cannot be directly analyzed. ChebNetII, a GNN model based on Chebyshev interpolation. Unlike full spectrum convolution, Chebnet avoids the eigenvalue decomposition of the Laplacian operator through recursive polynomial computation. It implements the k-order Chebyshev polynomial expansion of the spectral graph filter, which has faster convergence speed and reduces the Runge phenomenon when approximating the filter function. Which can be defined in Eq (2). Previous experimental studies have demonstrated that low-order polynomials are effective for function approximation, whereas higher order polynomials are required to capture more complex functional relationships [12].


$$\begin{array}{c} Y=\frac{2}{K+1}\sum_{\kappa =0}^{K}\sum_{j=0}^{K}\gamma_{j}\;T_{\kappa }(x_{j})T_{\kappa }(\hat{L})X \end{array}$$ (2)

In this formula, the parameter *K* denotes the order of Chebyshev polynomials, $\chi_{j}$ represents the Chebyshev nodes of $T_{K+1}$, and $\gamma_{j}$ denotes the learnable parameters.

#### Transformer layer.

In practical scenarios, the number of GNN layers is critical to model performance, as the limited depth of these networks constrains their capacity to capture multi-hop neighbors and global contextual information. This limitation is particularly pronounced when processing graph structured data with large scale and complex topologies, where the absence of multi-hop information often leads to degraded task performance. In contrast to GNNs, Transformer layers are capable of effectively modeling global information and multi-hop interactions through the multi-head attention mechanism, and they exhibit greater robustness to the oversmoothing problem commonly encountered in deep GNNs. The attention scores are computed using the scaled dot product formulation [13]. The multi-head attention mechanism performs multiple independent attention heads in parallel, and the resulting outputs are concatenated and subsequently transformed through a linear projection to produce the final output. The formulas are as follows Eqs (3) and (4).


$$\begin{array}{c} Q=HW^{Q},\;K=HW^{K},\;V=HW^{V} \end{array}$$ (3)

$$\begin{array}{c} \text{Attention}(Q,K,V)=\text{softmax}\left(\frac{QK^{T}}{\sqrt{d_{K}}}\right) \end{array}$$ (4)

The self-attention module projects the input features $H\in {\mathbb{R}}^{n\times d}$ into three subspaces, namely the query matrix *Q*, the key matrix *K*, and the value matrix *V*, where $W^{Q}\in {\mathbb{R}}^{d\times d_{K}}$, $W^{K}\in {\mathbb{R}}^{d\times d_{K}}$, and $W^{V}\in {\mathbb{R}}^{d\times d_{V}}$, with $d_{K}$ denoting the hidden dimension.

It is worth noting that the direct application of Transformers to graph data presents a significant challenge, as graph data contain both node features and structural topological information, with the latter being inherently difficult to encode as token sequences required by the Transformer architecture. To address this issue, existing approaches have primarily adopted two strategies [14]. The first strategy involves incorporating structural encoding modules directly into the Transformer, whereas the second strategy employs GNNs as auxiliary modules to preprocess graph structural information. The present study adopts the second strategy, utilizing ChebNetII as a graph structure preprocessor. Specifically, the input features are first passed through a ChebNetII layer, which aggregates local neighborhood information via K-order Chebyshev polynomial convolution, thereby encoding the graph structure into the node features. The enhanced features are then fed into the Transformer layer, enabling the Transformer to not only leverage its powerful global modeling capability but also indirectly perceive the graph topology through the input features. Furthermore, to strengthen the model’s ability to perceive graph structure, we introduce Laplacian positional encodings and random walk structural encodings as auxiliary information, which are injected into the network together with the original features. This approach effectively combines the inductive bias of GNNs in graph structure learning with the global interaction capacity of Transformers.

### Screening key genes

To accurately identify key genes in the “adenoma-cancer” transition of colorectal cancer, this study leverages the message passing mechanism of graph neural networks and the global capture capability of attention modules to replace traditional methods that rely on static centrality metrics, thereby dynamically learning the extent to which genes contribute to classification decisions. Following the learning of node representations by ChebTs, each gene is mapped to a value between 0 and 1 to compute its importance score, and the genes are subsequently ranked in descending order to generate a candidate gene list, which is defined as formula Eq (5). A higher score indicates more significant differential expression of that gene between the adenoma and carcinoma stages, suggesting its potential as a biomarker.


$$\begin{array}{c} S_{i}=\sigma (W_{1}h_{i}+b_{1}) \end{array}$$ (5)

Where $h_{i}\in {\mathbb{R}}^{128}$, *W*_1_ denotes learnable weight, and σ represents the sigmoid activation function.

### Functional enrichment analysis

To investigate the cellular pathways involved in adenocarcinoma transformation, the clusterProfiler package (version 4.16.0) was used to perform GO and KEGG enrichment analyses on the genes common to this process, yielding functional enrichment and pathway analyses of DEGs. GO annotations were derived from the GO.db package (GO release: 2025-02-06). In the GO analysis, enrichment was primarily presented in terms of biological processes (BP) [15]. KEGG analysis (KEGG release: 2026-05-31) was used to understand the functions and positions of DEGs in various pathways, particularly their roles in cancer related pathways.

### Validation of gene expression levels

Survival analysis was performed using K-M curves from the Kaplan-Meier Plotter to examine the impact of core gene expression on patients’ overall survival and relapse-free survival (RFS) [16]. Calculating the log-rank *P*, hazard ratio (HR), and 95% confidence interval (CI), a log-rank *P* < 0.05 was considered statistically significant.

### Virtual knockout

To systematically validate the necessity and causality of a given gene in the adenoma to carcinoma transition, we performed virtual knockout experiments at the system level to assess the effects of gene knockout on other genes. Given the practical constraints of experimental conditions and animal resources, systematic gene knockout experiments for a large number of genes remain challenging. We employed the scTenifoldKnk R package (version 1.0.2) for virtual knockout analysis. The gene co-expression network was constructed by calculating the Pearson correlation coefficient between genes, and the feature vector of the target gene in the adjacency matrix and the regulatory weight on the adjacent nodes are set to zero. Simulating the deletion of the target gene and comparing network topological changes before and after perturbation, the method screens for significantly affected downstream genes and pathways, thereby facilitating functional prediction of the target gene. Calculating the multiple changes of each gene to quantify the extent of its response to the perturbation of the target gene. ScTenifoldKnk has been demonstrated to be a robust and efficient approach for elucidating gene function, prioritizing knockout targets, and predicting experimental outcomes prior to conducting actual animal experiments [17].

## Results

### DEGs analysis and PPI network construction

All samples met the quality control criteria with values within the valid ranges. Differential expression analysis revealed 2392 significantly dysregulated genes between normal mucosa and adenoma tissues, of which 717 were upregulated and 1675 were downregulated. Between adenoma and colorectal carcinoma tissues, 228 significant genes were identified, comprising 109 upregulated and 119 downregulated genes. Between normal mucosa and colorectal carcinoma tissues, 2749 significant genes were detected, with 1019 upregulated and 1730 downregulated (Fig 2).

**Fig 2:**
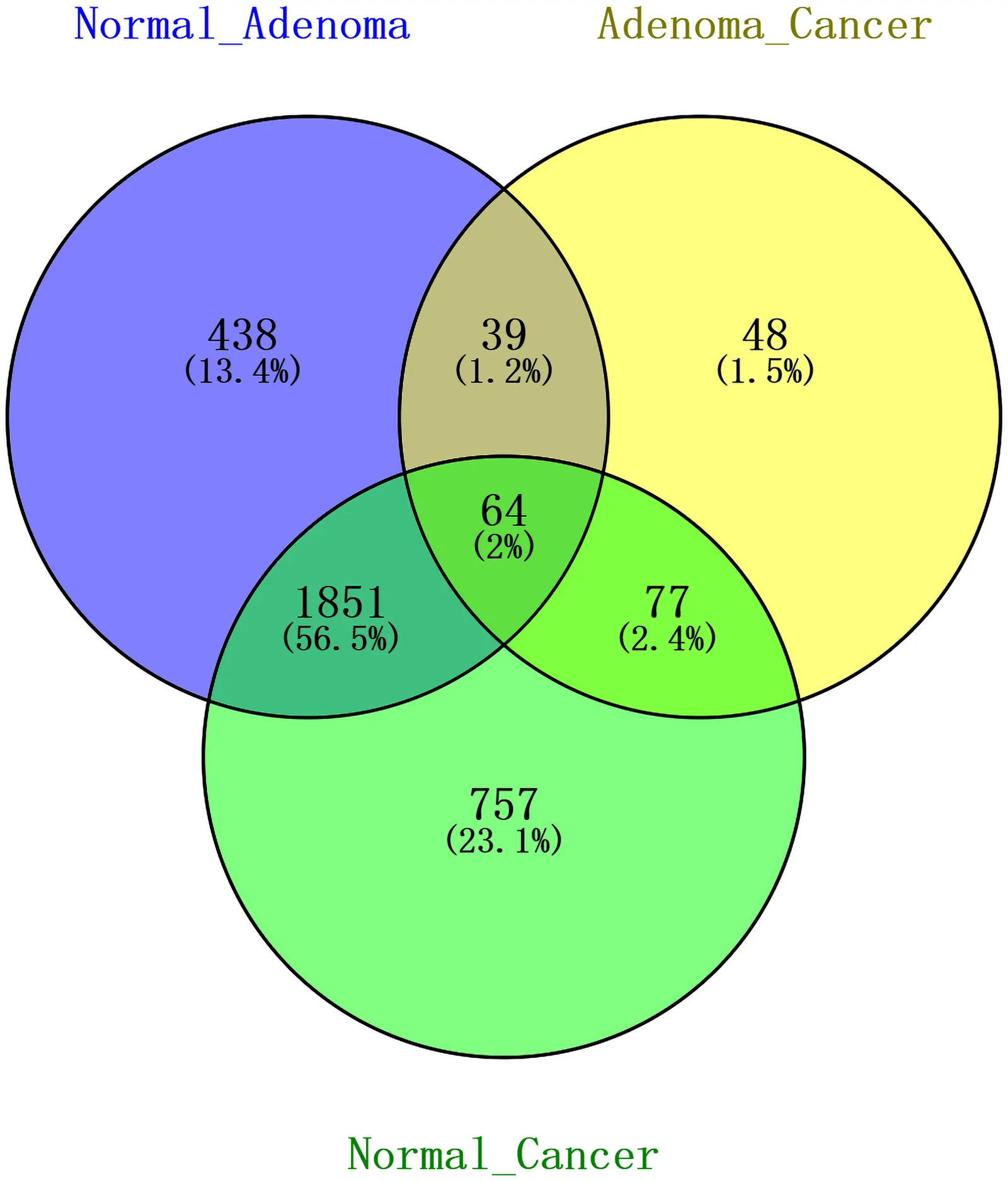
Venn graph. The overlapping parts of the two circles represent the common genes between the two datasets. A Venn diagram reveals 64 genes that were consistently dysregulated throughout the entire transformation sequence.

Using the Heatmap package, we generated a clustering analysis heatmap for the top 30 DEGs (Fig 3).The three types of samples show significant differences in their gene expression profiles, which can effectively distinguish different pathological stages. The fold change (FC) in expression on the x-axis and the magnitude of expression change on the y-axis, Fig 4A and 4B shows a volcano plot of the DEGs to illustrate the degree of difference and statistical significance [10]. DEGs were analyzed using the STRING database with a confidence score greater than 0.4 [9]. The top 300 genes were selected and imported into Cytoscape software for visualization of the results (Fig 5).

**Fig 3:**
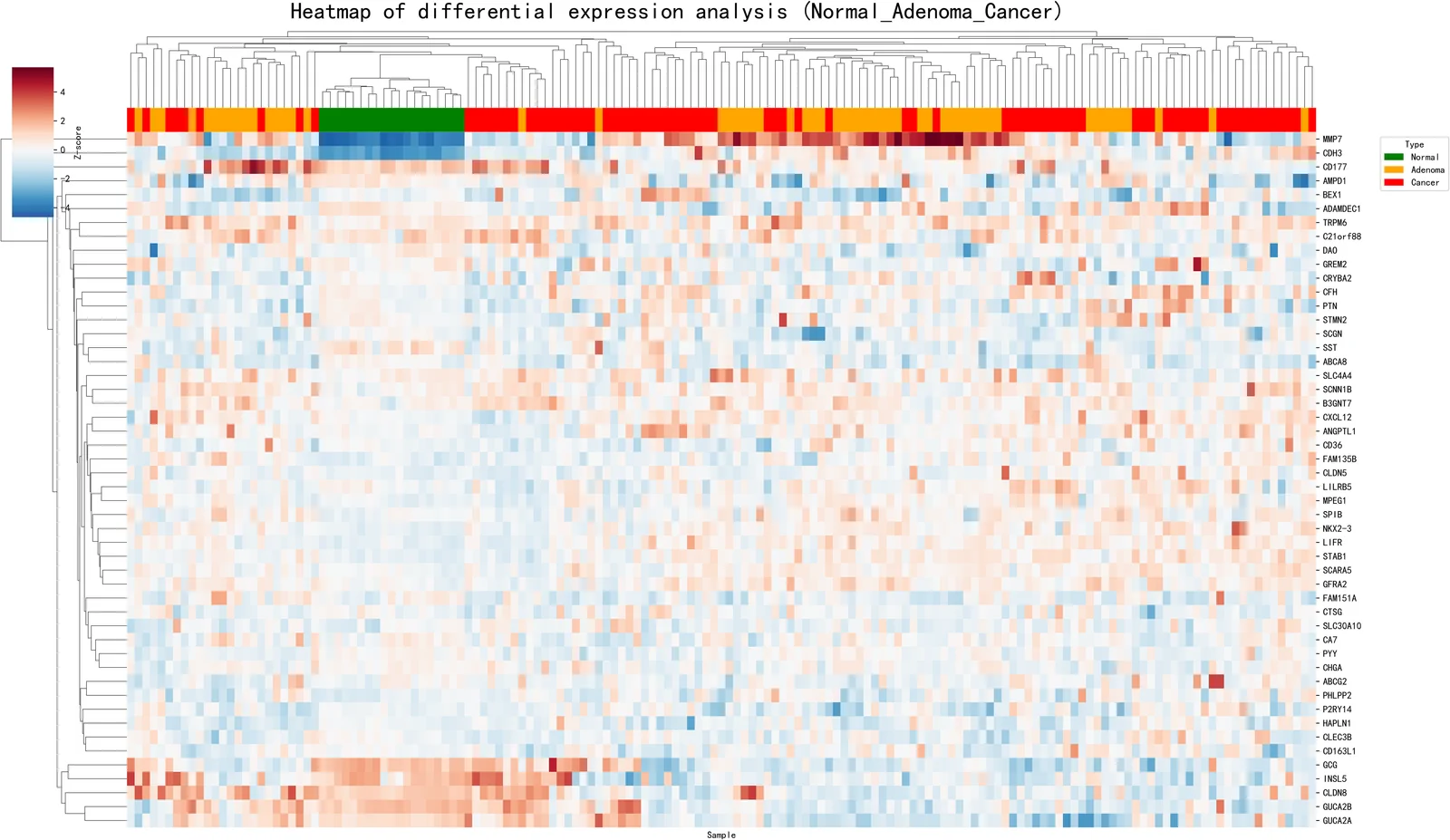
Heatmap of the top 30 DEGs involved in the entire sequence transformation. The x-axis represents different samples, while the y-axis indicates different gene names. Green indicates the normal stage of the samples, yellow indicates adenoma, and red indicates cancer. The shade of color reflects the level of expression, red indicates high expression, and blue indicates low expression.

**Fig 4:**
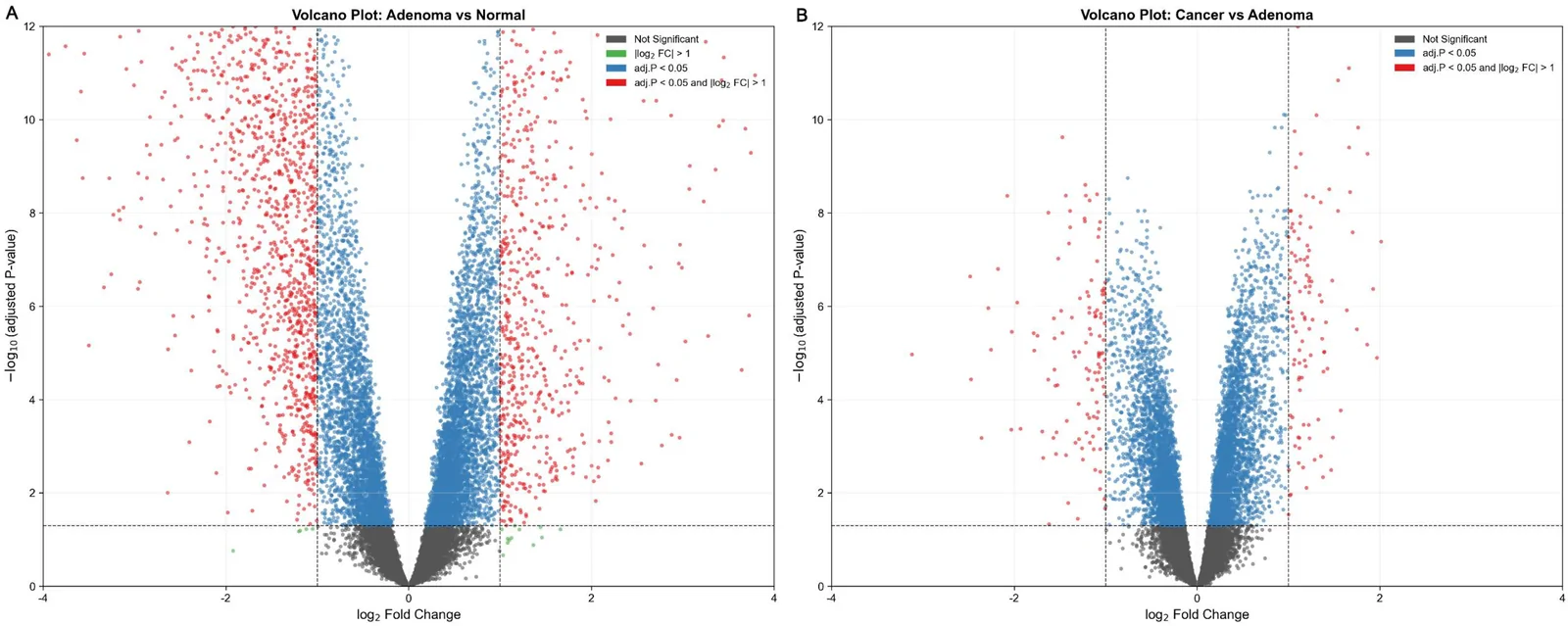
Volcano plots of differential expressed genes. A: Volcano plot of DEGs between adenoma and normal. B: Volcano plot of DEGs between cancer and adenoma. Red indicates significantly different genes. The right side represents upregulated genes, the left side represents down-regulated genes. Blue indicates statistically significant genes with small change amplitudes. Green indicates genes with large change amplitudes but not significant. Gray indicates genes without significant differences.

**Fig 5:**
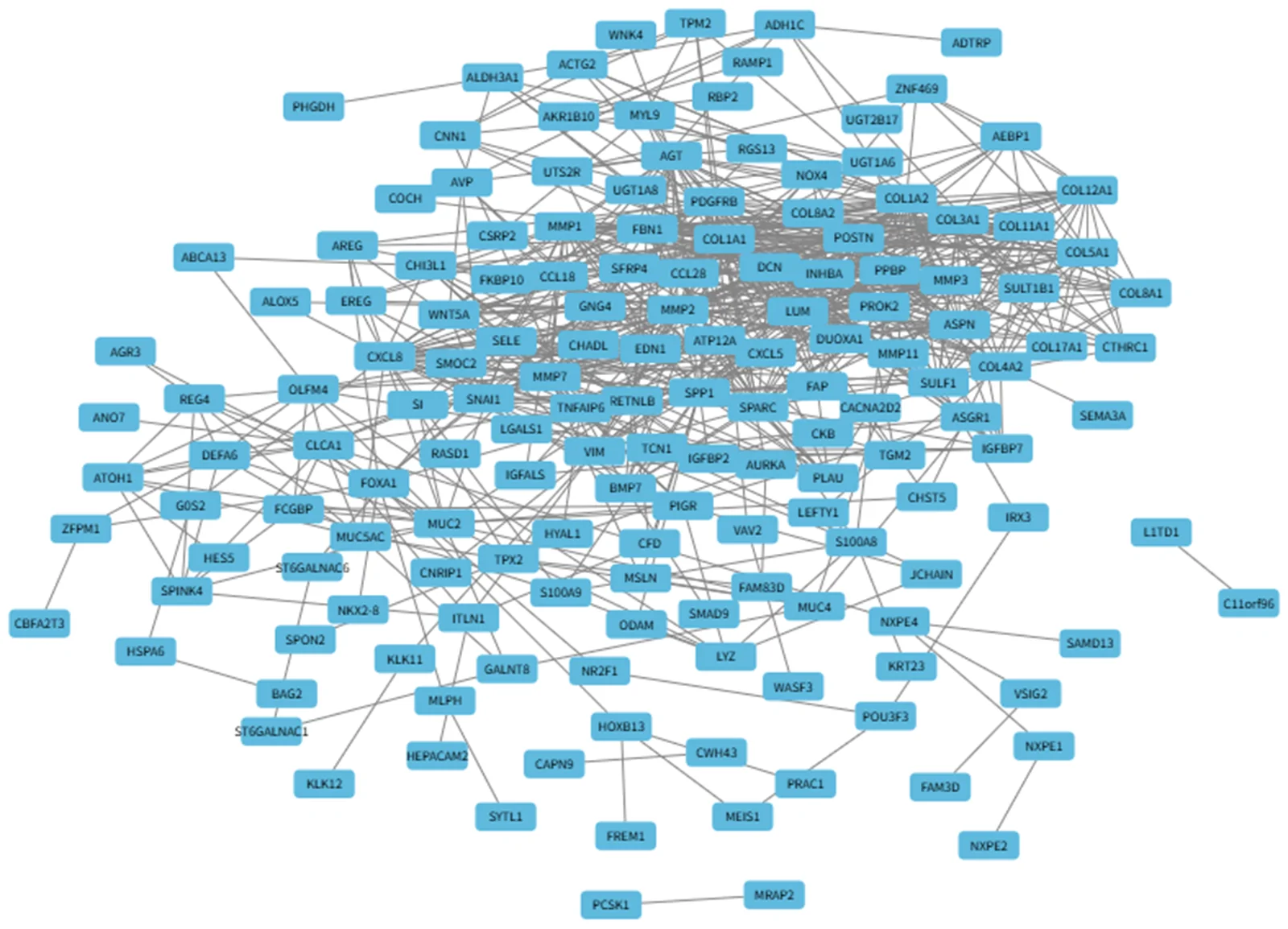
PPI network. A total of 1,293 protein nodes and 2,423 interaction relationships formed.

### Evaluation indicators

To evaluate the capacity of the proposed model for predicting key genes, a multi-level evaluation framework was adopted, comprising three metrics, the area under the receiver operating characteristic curve (AUROC), the area under the precision-recall curve (AUPRC), and the F1-score [18]. In the confusion matrix [19], True Positive (TP) refers to the number of correctly predicted key genes, True Negative (TN) refers to the number of correctly predicted non-key genes, False Positive (FP) refers to the number of incorrectly predicted key genes, and False Negative (FN) refers to the number of in-correctly predicted non-key genes. The calculation formulas are as follows:


$$\begin{array}{c} Precision=\frac{TP}{TP+FP} \end{array}$$ (6)

$$\begin{array}{c} Recall=\frac{TP}{TP+FN} \end{array}$$ (7)

$$\begin{array}{c} F1=2\times \frac{Precision\times Recall}{Precision+Recall} \end{array}$$ (8)

In the formula Eq (9), $N_{i}$ represents the number of positive samples, and $N_{j}$ represents the number of negative samples. *I* denotes an indicator function. It takes the value of 1 when the condition is satisfied, and 0 otherwise. *P* represents the predicted score of the sample.


$$\begin{array}{c} AUC=\frac{\sum_{i}\sum_{j}I(P_{i}>P_{j})}{N_{i}\times N_{j}} \end{array}$$ (9)

*P*(*r*) denotes the precision of the model at recall value *r* after smoothing. We obtain the approximate area (AP) under the P-R curve by integration [18], which is defined as formula Eq (10).


$$\begin{array}{c} AUPRC=\int_{0}^{1}P(r)\;dr \end{array}$$ (10)

### Baseline comparison experiment

Under otherwise identical experimental conditions, we conducted a comparative analysis of ChebTs against the baseline models. As shown in Table 1, our analysis yielded three main findings. First, ChebTs outperformed all other models across all evaluation metrics, achieving an F1-score of 0.8831, which indicates a marked advantage in handling class imbalance in the dataset. Furthermore, both AUROC (0.9432) and AUPRC (0.9593) achieved optimal values, revealing that this model offers higher reliability in identifying key genes. Second, the results validate the effectiveness of Chebyshev convolutional neural networks in this context. Previous studies have shown that ChebNet consistently outperforms traditional GCNs across various graph structured learning tasks [8]. This superiority is attributed to the fact that Chebyshev polynomials approximate spectral filters via polynomial expansion, which enables more effective local feature extraction while reducing computational complexity, and allows better capture of topological information from multi-hop neighbors without the computational bottleneck imposed by eigenvector decomposition in conventional spectral methods. Third, it highlights the advantages of graph structural information. By fusing the spectral domain of Chebyshev convolution with the global attention mechanism of Transformers, ChebTs achieves results significantly superior to those of traditional machine learning, demonstrating that the interactive fusion of graph structural information and higher order features can effectively enhance classification performance. In summary, the proposed ChebTs model, which leverages both the topological structure of interaction networks and original expression features, provides an effective approach for identifying diagnostic biomarkers in CRC.

**Table 1:** Comparison experiment table.

Method	AUROC	AUPRC	F1-score	*P*-value
RF	0.8693	0.8742	0.8107	< 0.001
SVM	0.8977	0.9142	0.8466	< 0.001
GAT	0.8618	0.8944	0.7706	< 0.001
GCN	0.7764	0.8251	0.6720	< 0.001
ChebNet	0.8898	0.9315	0.7806	< 0.001
EMOGI	0.9046	0.9284	0.7905	< 0.001
Cheb+Trans+PESE	0.9062	0.9395	0.6556	< 0.001
ChebTs (Ours)	0.9432	0.9593	0.8831	–

### Ablation experiment

The ChebTs model was developed on basis of ChebNet. To further validate the effectiveness of each component, we designed ablation experiments, with the results summarized in Table 2. The removal of any single feature consistently led to a decrease in all evaluation metrics, indicating that the combination of biological features with network structural information enhances model predictive performance. Notably, the removal of the attention mechanism resulted in a decrease in AUROC of approximately 1.20%, a decrease in AUPRC of approximately 1.15%, and a decrease in the F1-score of nearly 1.39%, underscoring the critical role of multi-head attention in feature fusion. Furthermore, the removal of both PE and SE led to a substantial reduction in AUROC and F1-score, suggesting that feature encodings contribute significantly to model performance. Previous studies have demonstrated that random walk structural encodings capture local topological features, while Laplacian positional encodings provide spatial structural information for graph nodes, thereby compensating for the inherent insensitivity of GNNs to node ordering [11]. The ablation results confirm the important role of feature encodings in network architecture. Collectively, ChebNetII, the Transformer branch, and the PE/SE encoding modules work synergistically and are all indispensable components of ChebTs. Through multi-feature learning, the model achieves accurate classification of colorectal adenomas and carcinomas, further validating the effectiveness of integrating spectral graph convolution with the Transformer attention mechanism. The ROC and P-R curves comparing ChebTs with baseline models are presented in Figs 6 and 7.

**Table 2:** Ablation experiment table.

Method	AUROC	AUPRC	F1-score
Only ChebNetII	0.7332	0.8012	0.6083
Without Transformer	0.7859	0.8333	0.6350
Without PE/SE	0.8844	0.9316	0.7750
ChebTs (Ours)	0.9432	0.9593	0.8831

**Fig 6:**
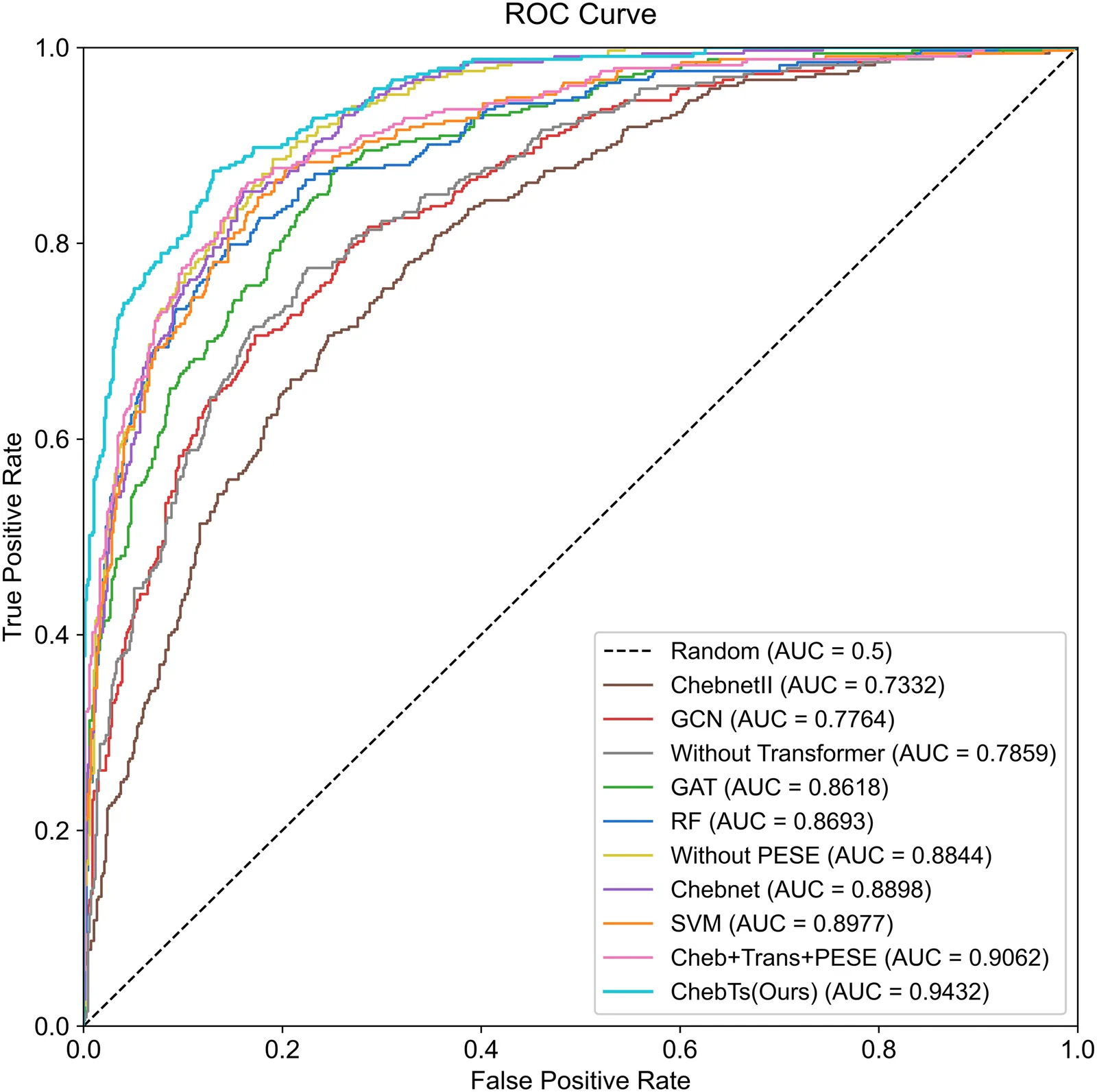
ROC curves of different methods on the colorectal cancer dataset.

**Fig 7:**
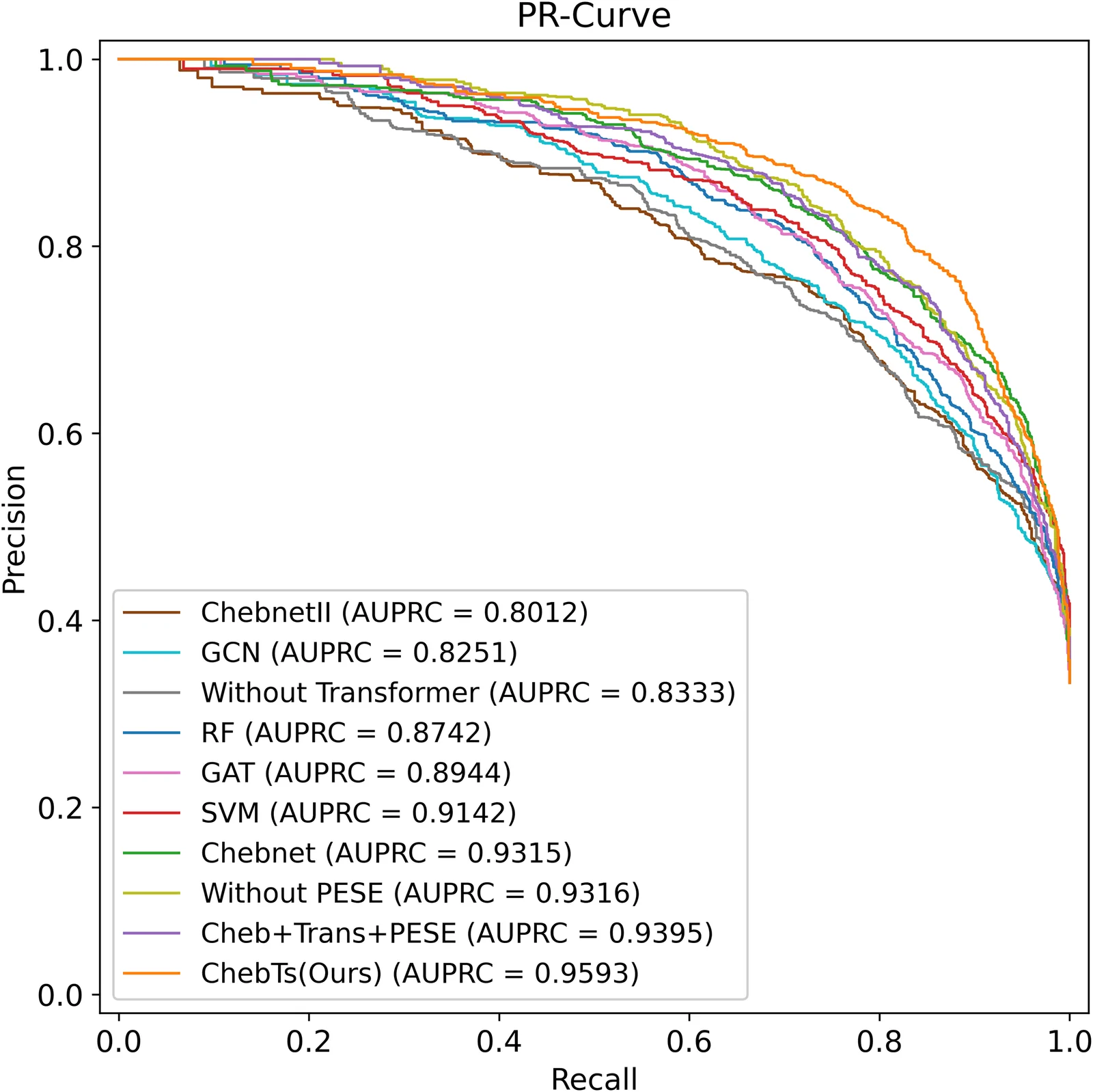
P-R curves of different methods on the colorectal cancer dataset.

### Enrichment analysis and functional annotation

GO enrichment analysis was performed on the DEGs to identify associated biological processes. As shown in Fig 8, the enrichment results were significantly concentrated in extracellular matrix (ECM) related processes, including extracellular matrix organization, extracellular structure organization, and external encapsulating structure organization, with high statistical significance. Notably, the prominent enrichment of ECM related pathways suggests that ECM remodeling may represent a critical event in the malignant transformation of colorectal cancer. The ECM is a core component of the tumor microenvironment, and aberrant ECM remodeling has been established as a key driver of tumor progression, invasion, metastasis, and therapeutic resistance [20]. Excessive ECM deposition and cross linking can form physical barriers that limit drug penetration and promote epithelial mesenchymal transition (EMT) in tumor cells, whereas dysregulated collagen metabolism further exacerbates fibrosis and immunosuppression within the tumor microenvironment [21–23].

**Fig 8:**
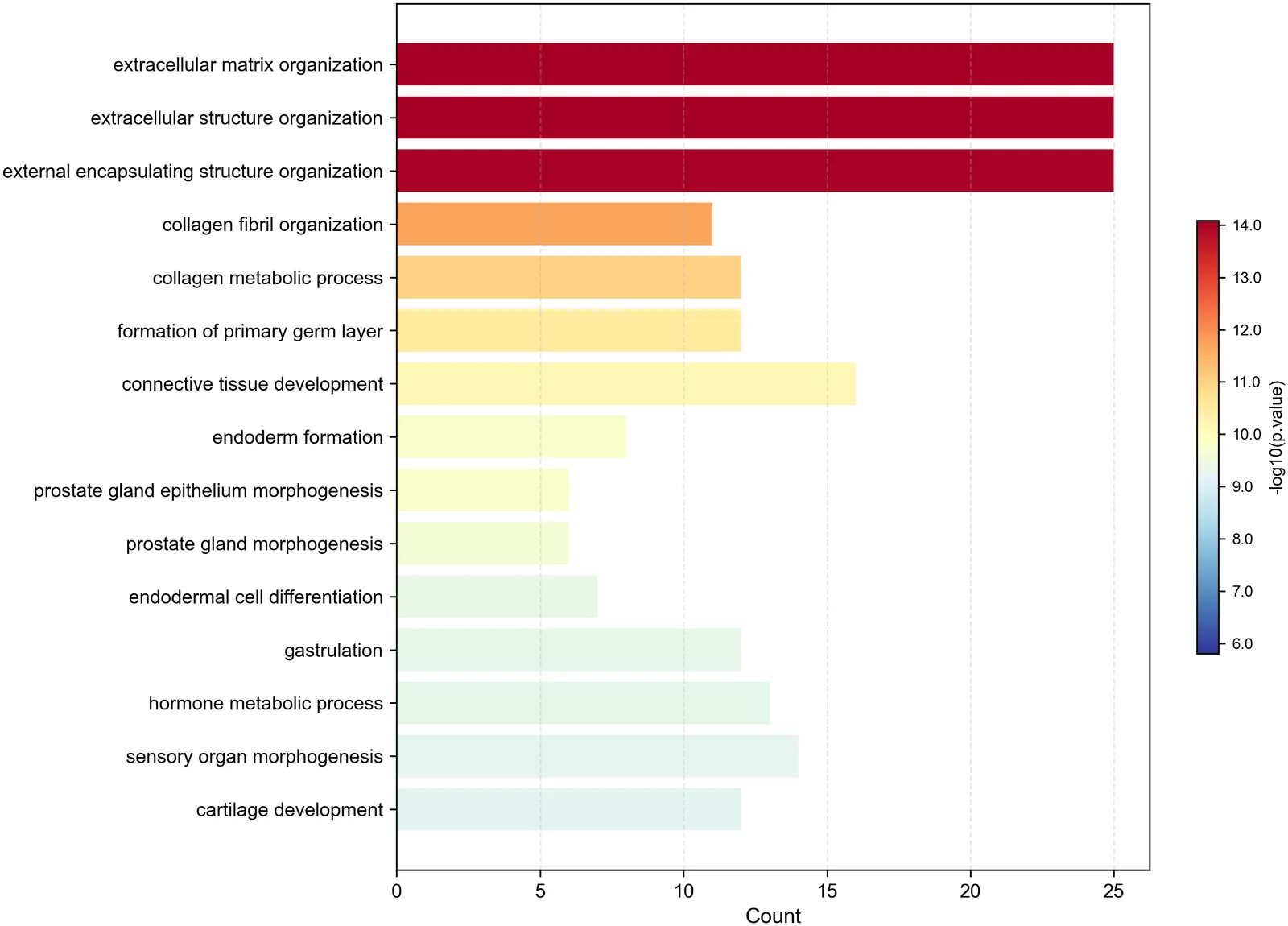
Enrichment analysis of GO function pathway.

KEGG pathway enrichment analysis further revealed the signaling pathways involved in the adenoma to carcinoma transition, with significant enrichment observed in pathways related to metabolic disorders, inflammatory regulation, and the tumor microenvironment. Among these, pathways including protein digestion and absorption, the AGE-RAGE signaling pathway, and the regulation of muscle cell cytoskeleton were found to be significantly enriched. Metabolic reprogramming is recognized as one of the core hallmarks of cancer. Aberrant protein metabolism not only supplies the essential substrates and energy required for rapid tumor cell proliferation but also contributes to tumor progression by modulating nutrient allocation within the tumor microenvironment and suppressing immune cell function [24,25].

The AGE-RAGE signaling pathway plays a critical role in the crosstalk between chronic inflammation and the tumor microenvironment. Obesity-related adipose tissue dysfunction can lead to elevated levels of inflammatory cytokines, such as IL-6 and TNF-α, which in turn promote ECM remodeling [26]. This finding is consistent with the GO enrichment results obtained in the present study. Furthermore, the enrichment of the ECM receptor interaction pathway further revealed that this pathway mediates bidirectional signaling between cells and the ECM. As the primary receptors for ECM components, integrins, upon aberrant activation, can trigger the FAK/Src signaling cascade, thereby regulating cell proliferation, survival, and migration [20]. Studies have demonstrated that the interaction between collagen and integrins can promote EMT in tumor cells, which represents a critical step in the acquisition of invasive and metastatic capabilities [26]. In addition, colon cancer cells have been shown to induce de novo glycine synthesis in cancer-associated fibroblasts (CAFs) through the secretion of TGF-β1, thereby promoting collagen production [27]. This observation suggests a metabolic crosstalk between tumor cells and stromal cells, forming a positive feedback loop between ECM remodeling and TGF-β signaling that collectively drives tumor progression [28]. The enrichment results are presented in Fig 9.

**Fig 9:**
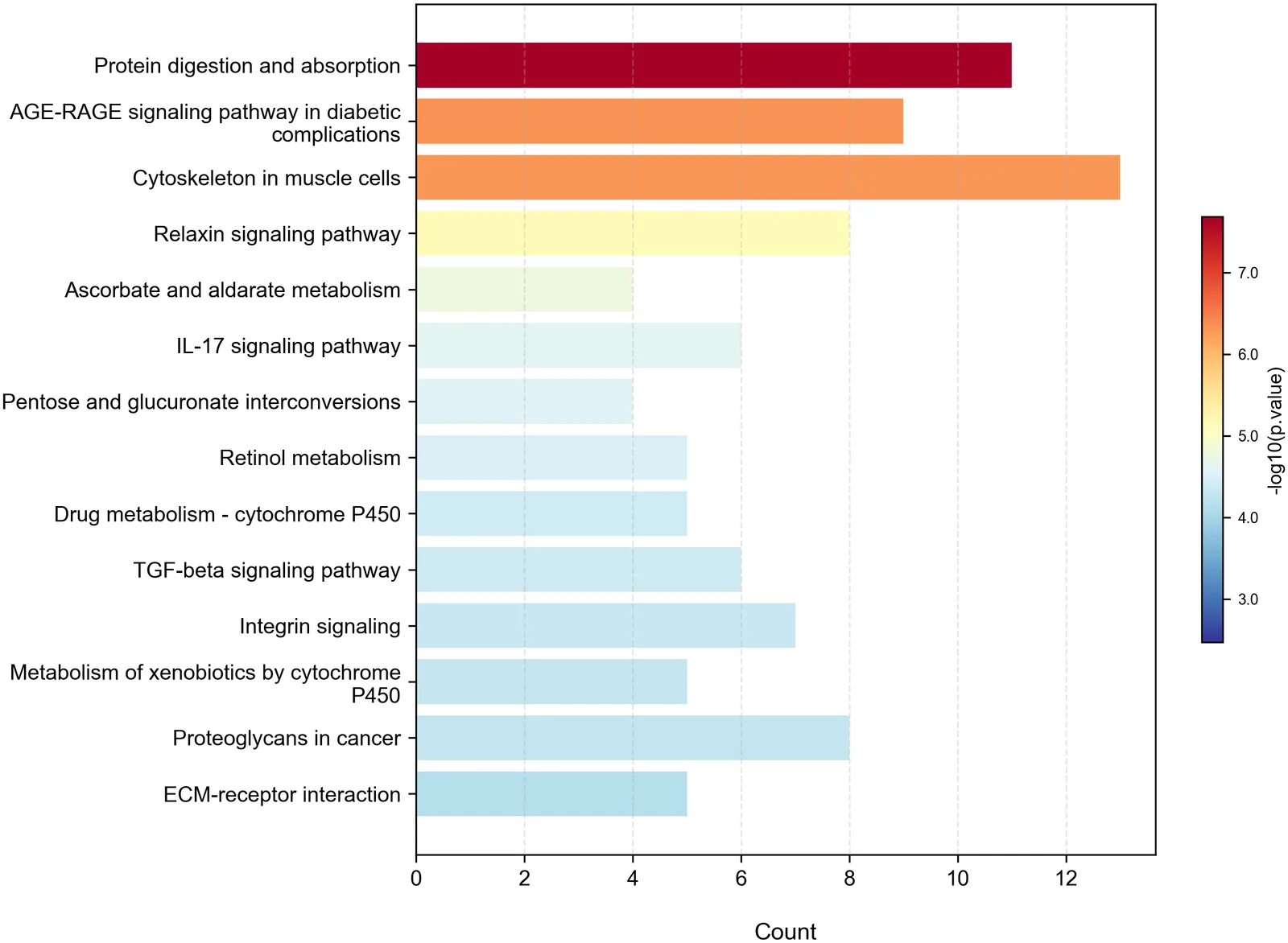
Enrichment analysis of KEGG pathway.

### Comparison of key gene performance of ChebTs in predicting different types of cancer

To evaluate the predictive performance of the model on other cancer types, we selected two independent datasets for liver cancer and pancreatic cancer as test sets. Datasets were processed using the same pipeline as the CRC dataset, with genes serving as nodes and PPI networks as edges. After incorporating the encoded features, the data were input into ChebTs for training. Notably, no hyperparameter tuning was performed on these two disease datasets, while all other data processing procedures and methodological settings were kept consistent with those used for CRC. The results are presented in Table 3. We observed that small sample sizes and class imbalance tended to impair the model’s discriminative ability, whereas relatively balanced sample distributions were more favorable for class feature determination. Nevertheless, our model demonstrated superior stability and robustness.

**Table 3:** Comparison table of performance of different cancer prediction genes.

Tumor Type	Dataset	T/NT Size	AUROC	AUPRC	F1-score
Colorectal Cancer	GSE31905	80/56	0.9432	0.9593	0.8831
	GSE41657				
Liver Cancer	GSE54236	81/80	0.9688	0.9711	0.8730
Pancreatic Cancer	GSE62452	60/61	0.9062	0.9060	0.8195

Among the top 10 genes identified by ChebTs, seven have been validated by literature evidence as diagnostic biomarkers for hepatocellular carcinoma. These genes are listed in Table 4 and include *HOXA13*, *NPC1L1*, *TDO2*, *AKR1B10*, *APOF*, *ADH1B*, and *CYP2C19*. In addition, six genes associated with pancreatic cancer were identified as prognostic biomarkers, as shown in Table 5, *MUC17*, *MBOAT2*, *SLC6A14*,*MUC13*, *ANXA10*, and *GALNT5*. These genes may influence cancer prognosis or participate in tumor related cellular activities, positioning them as key molecular players in cancer progression [29]. The discovery of these biomarkers may offer potential opportunities for inhibiting tumor advancement or enhancing the efficacy of current therapeutic strategies.

**Table 4:** The literature basis for ChebTs’ prediction of key genes for liver cancer.

Gene	Function
*HOXA13*	High expression of the *HOXA13* gene is an important marker of poor prognosis in patients with hepatocellular carcinoma. It provides new insights into the development of liver cancer and can serve as a biomarker [30].
*NPC1L1*	*NPC1L1* is a cholesterol transporter primarily expressed in the intestine and liver. It is significantly downregulated in hepatocellular carcinoma tissues, which may influence tumor growth and immune evasion [31].
*TDO2*	*TDO2* is positively correlated with the pathological characteristics and prognosis of hepatocellular carcinoma, and promotes cell migration, invasion, and proliferation [32].
*AKR1B10*	*AKR1B10* has been identified as a key prognostic biomarker associated with the histone lactylation pathway in hepatocellular carcinoma [33].
*APOF*	Downregulated *ApoF* expression in hepatocellular carcinoma is associated with lower recurrence-free survival, it may act as a tumor suppressor [34].
*ADH1B*	*ADH1B* is considered an independent factor in improving the survival prognosis of patients with hepatocellular carcinoma, and its high expression is significantly associated with a favorable survival rate [35].
*CYP2C19*	*CYP2C19* may serve as a potential prognostic marker for hepatocellular carcinoma following liver resection [36].

**Table 5:** The literature basis for ChebTs’ prediction of key genes for pancreatic cancer.

Gene	Function
*MUC17*	*MUC17* may serve as a novel molecular prognostic biomarker associated with lymph node metastasis in pancreatic ductal adenocarcinoma [37].
*MBOAT2*	*MBOAT2* may serve as a potential therapeutic target for pancreatic cancer; its high expression is closely associated with tumor aggressiveness and poor prognosis [38].
*SLC6A14*	*SLC6A14*-mediated glutamine promotes SYTL4-CXCL8 axis activation to drive immune evasion in pancreatic cancer [39].
*MUC13*	*MUC13*, a protein specific to pancreatic ductal adenocarcinoma, drives tumor progression, metastasis, and immune evasion through its complex network of protein interactions [40].
*ANXA10*	*ANXA10* can serve as a biomarker for the diagnosis of early-stage pancreatic ductal adenocarcinoma [41].
*GALNT5*	*GALNT5* functions as a suppressor of ferroptosis and a potential predictor of poor prognosis in pancreatic adenocarcinoma [42].

### Gene interpretable analysis based on GNNExplainer

GNNExplainer identifies the most influential node features and subgraph structures for classification tasks by optimizing a mask mechanism. To elucidate the decision making process of ChebTs in identifying disease relevant genes, we performed interpretability analysis on the eight candidate genes, and the results are summarized in Table 6. Based on the GNNExplainer analysis of the DEGs, *MMP1*, *STC2*, *NOX4*, and *IL-33* were found to be consistently upregulated across both stages, suggesting that these genes exert oncogenic functions during colorectal cancer progression and may represent potential cancer driver genes. In contrast, *SFRP1* was persistently downregulated, indicating its role as a tumor suppressor gene. *CLDN2* exhibited an upregulation in the normal to adenoma stage followed by downregulation in the adenoma to carcinoma stage, suggesting its potential utility as an adenoma specific biomarker. *FN1* and *IGFBP7* displayed a reversed expression pattern, with downregulation at the adenoma stage and upregulation at the carcinoma stage, indicating their potential value as prognostic biomarkers for CRC. According to the gene importance scores, further highlighting the central role of *FN1* in cancer progression. Collectively, these findings indicate that malignant transformation from adenoma to carcinoma involves multi-gene expression alterations. These molecules not only serve as early diagnostic biomarkers for colon cancer but also provide unique insights into the molecular mechanisms underlying the transition from precancerous lesions to malignancy.

**Table 6:** GNNExplainer of genes.

Rank	Gene	Adenoma-Cancer	Normal-Adenoma	Importance score	Predicted class
1	*FN1*	Up	Down	0.9729	Cancer
2	*CLDN2*	Down	Up	0.9398	Adenoma
3	*IL-33*	Up	Up	0.9315	Adenoma
4	*MMP1*	Up	Up	0.9297	Cancer
5	*STC2*	Up	Up	0.9151	Cancer
6	*IGFBP7*	Up	Down	0.9010	Cancer
7	*NOX4*	Up	Up	0.8569	Cancer
8	*SFRP1*	Down	Down	0.8194	Adenoma

To further elucidate the synergistic regulatory relationships among the identified genes, we calculated Pearson correlation coefficients for the eight core genes based on gene expression data across all samples and constructed a correlation heatmap (Fig 10). The analysis revealed complex network regulatory relationships among these genes. Specifically, *NOX4* and *MMP1* exhibited a significant positive correlation (r = 0.50). Consistent with this finding, previous studies have demonstrated that knockout of *NOX4* leads to a marked reduction in *MMP1* gene expression levels [43], suggesting that these two genes may act synergistically in the regulation of oxidative stress and the remodeling of the tumor microenvironment. In contrast, *SFRP1* and *STC2* showed a significant negative correlation (r = −0.25), revealing a dual mechanism in colon cancer progression, in which inactivation of a tumor suppressor gene and activation of a pro-oncogenic gene occur concurrently. In colon cancer, *SFRP1* is frequently downregulated due to promoter hypermethylation, leading to aberrant activation of the Wnt signaling pathway [44] and thereby promoting tumor initiation. Meanwhile, *STC2* facilitates tumor cell migration by participating in EMT [45]. Together, these two genes synergistically promote the malignant progression of colorectal cancer through distinct signaling pathways. Interpretable analysis using GNNExplainer revealed that these genes are involved in multiple cancer related biological processes, including extracellular matrix remodeling (*MMP1*, *FN1*), tight junction disruption (*CLDN2*), inflammatory immune modulation (*IL-33*), oxidative stress (*NOX4*), and aberrant Wnt signaling (*SFRP1*).

**Fig 10:**
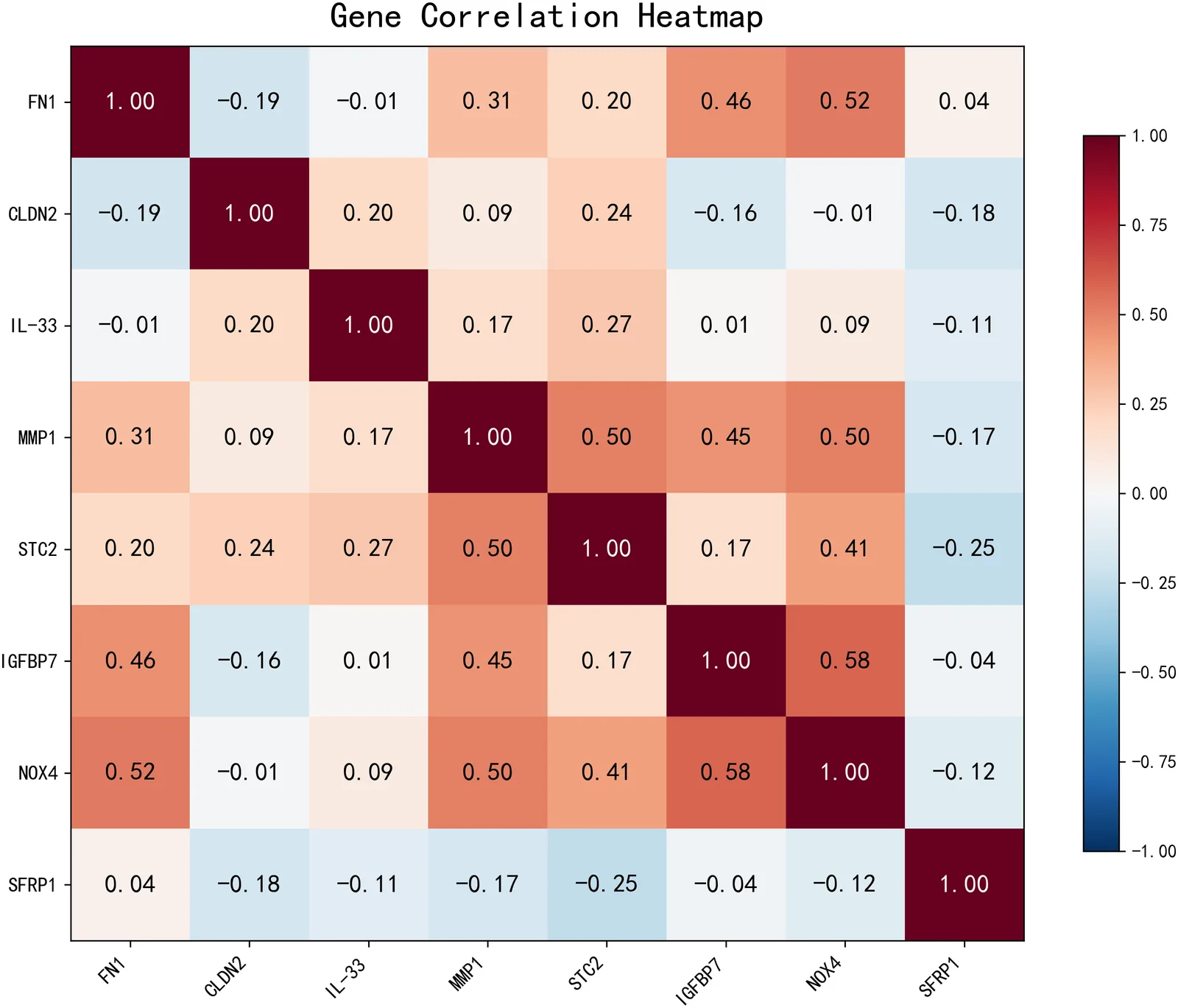
Heatmap of gene related correlation. The gene correlation among eight key genes are calculated by Pearson correlation coefficient. The redder the color, the higher the correlation coefficient, while the bluer the color, the lower the correlation coefficient.

## Verification

### Survival prognosis analysis of core genes

To evaluate the prognostic value of the eight key genes, we performed survival analysis using Kaplan-Meier Plotter, and the results are presented in Fig 11. The eight genes, *FN1*, *CLDN2*, *IL-33*, *MMP1*, *STC2*, *IGFBP7*, *NOX4*, and *SFRP1*, included six oncogenes and one tumor suppressor gene. Furthermore, we found that *IGFBP7* exhibits oncogenic functions in paracrine tumor stroma interactions but displays tumor suppressive functions when inactivated by DNA methylation [46]. *IGFBP7* has also been identified as a TGF-β target gene and a tumor-stroma marker in epithelial cancers, and its high expression has been significantly associated with overall survival in colorectal cancer patients.

**Fig 11:**
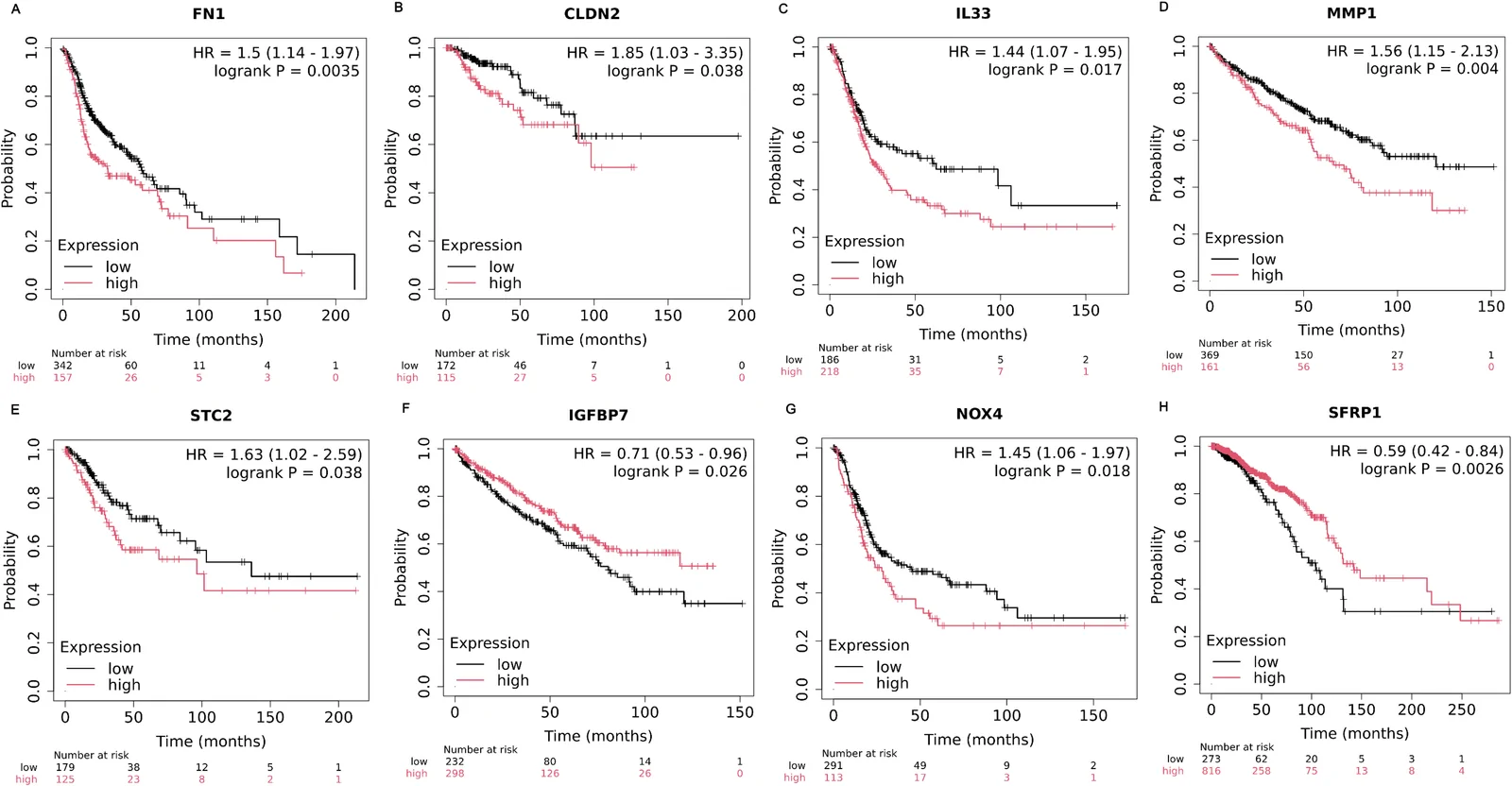
Kaplan-Meier Plotter survival analysis of hub genes. The x-axis represents time in months, and the y-axis represents the survival rate of the patients. The red line represents patients with high expression, while the black line represents patients with low expression. A-H: Survival curves for individual genes, including A: *FN1*, B: *CLDN2*, C: *IL-33*, D: *MMP1*, E: *STC2*, F: *IGFBP7*, G: *NOX4*, and H: *SFRP1*.

Among these, the *IL-33* receptor ST2 is highly expressed in tumor associated macrophages, and this has been shown to correlate significantly with poor survival and reduced cytotoxicity of CD8 + T cells in colorectal cancer patients, suggesting that the *IL-33*/ST2 signaling axis promotes tumor progression by shaping an immunosuppressive microenvironment [47]. *MMP1* [48] and *CLDN2* [49] are both highly expressed in colorectal cancer and are associated with poor prognosis. *SFRP1* is epigenetically silenced through promoter methylation and functions as a tumor suppressor [50]. *NOX4* has demonstrated significant prognostic value in left-sided colon cancer, with high expression indicating an elevated risk of cancer development [51]. Knockout of *STC2* has been shown to suppress malignant phenotypes in colon cancer [52], and high expression of *FN1* has been found to correlate significantly with tumor progression and poor prognosis [53].

### Verification of virtual knockout for analysis of perturbation effects of key genes

To assess the regulatory impact of the eight core genes identified in this study, each gene was individually subjected to virtual knockout analysis, and differentially regulated genes were subsequently identified using manifold alignment. The analysis was performed with the following parameter settings: single core processing, qc = TRUE, nc_nCells = 50, and nc_nNet = 5. The FC values for each sequential gene knockout are compared in Fig 12. The results revealed that knockout of each of the eight genes led to significant perturbations in the regulatory network, and each knockout condition generated a specific set of differentially regulated genes. Among the eight genes evaluated, knockout of *IL-33* elicited the most extensive downstream effects, indicating that *IL-33* exerts a prominent regulatory influence within the gene network governing colorectal tumorigenesis. To validate the reliability of the virtual knockout predictions, we randomly selected a panel of genes for knockout and calculated their FC values. The upper bound of the 95% confidence interval for the random knockout group was 0.82, whereas the effect sizes of the target genes were substantially higher than this threshold. Collectively, these results suggest that the gene perturbation effects identified in our study are characterized by high specificity.

**Fig 12:**
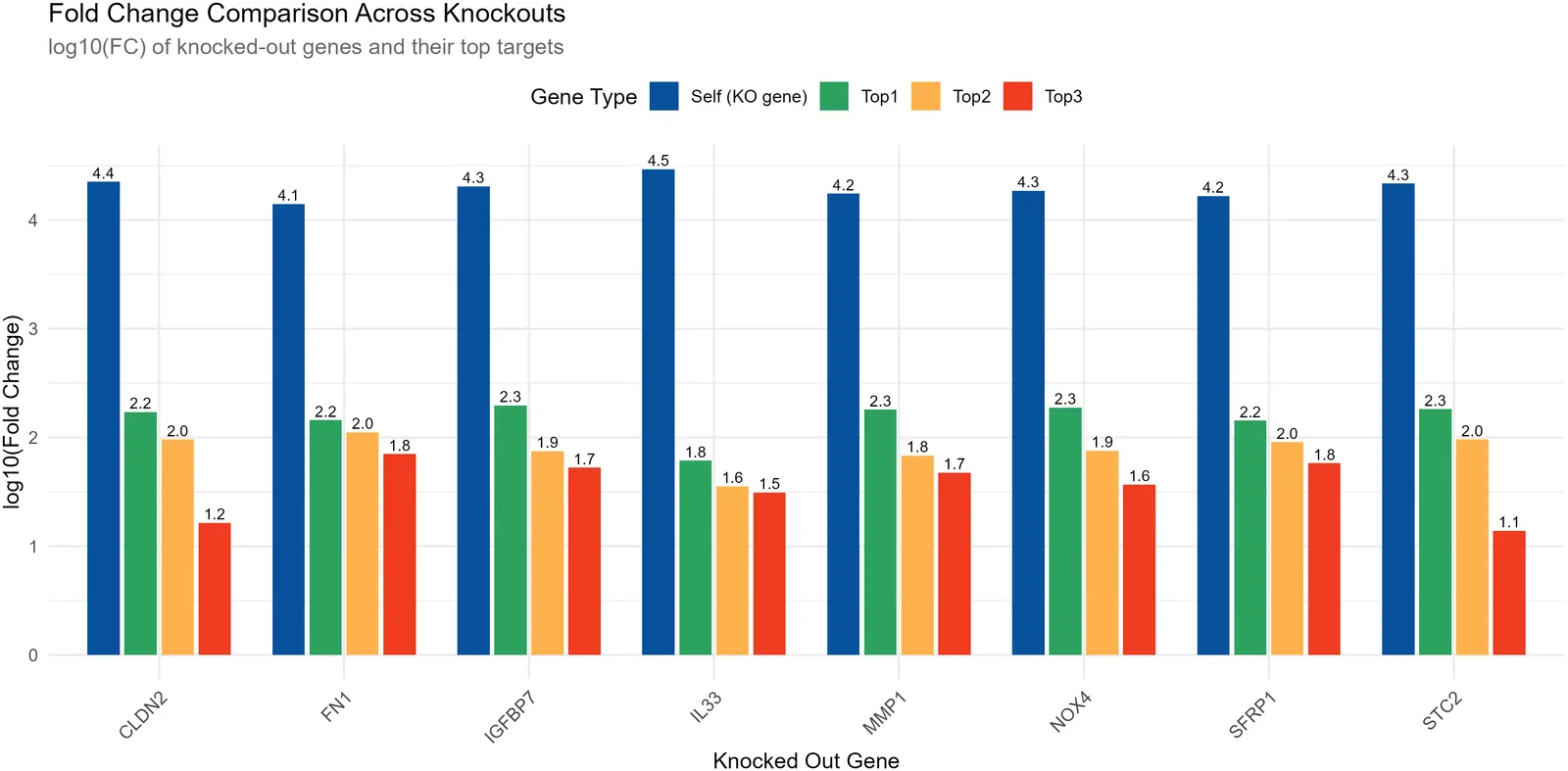
FC Value comparison graph. The highest FC value in each knockout experiment was consistently attributed to the knocked-out gene itself, confirming the validity of the virtual knockout approach.

According to the heatmap of common target genes (Fig 13), the palmitoyltransferase encoding gene *ZDHHC9* exhibited significant differential expression across all eight knockout groups, with the strongest effects observed following the knockout of *MMP1*, *NOX4*, and *STC2*, suggesting that it may be a key downstream effector in the process of carcinogenesis. Studies have shown that *ZDHHC9* promotes the development of colorectal cancer by mediating the palmitoylation of *KLF5*, thereby enhancing signaling pathways such as cAMP [54], suggesting that *ZDHHC9* may serve as a compensatory mechanism in tumor cells in response to genetic perturbations. To identify core genes that were consistently responsive to the knockout of the eight genes, we analyzed the occurrence frequencies of the top four upregulated genes. In addition to *ZDHHC9*, the results revealed that *TRIB3* (7/8), *TOP1* (6/8), and *CWC25* (5/8) also appeared frequently across multiple knockout conditions. These responsive genes could be classified into two functional modules. The first module is associated with immune stress and includes *TRIB3*, which is involved in stress sensing and autophagy regulation, and was predominantly activated following the knockout of *IL-33*, *CLDN2*, and *STC2* [55]. The second module is related to proliferation and DNA repair and includes *TOP1*, a DNA topoisomerase, which was mainly activated following the knockout of *MMP1*, *NOX4*, *SFRP1*, *FN1*, and *IGFBP7* [56].

**Fig 13:**
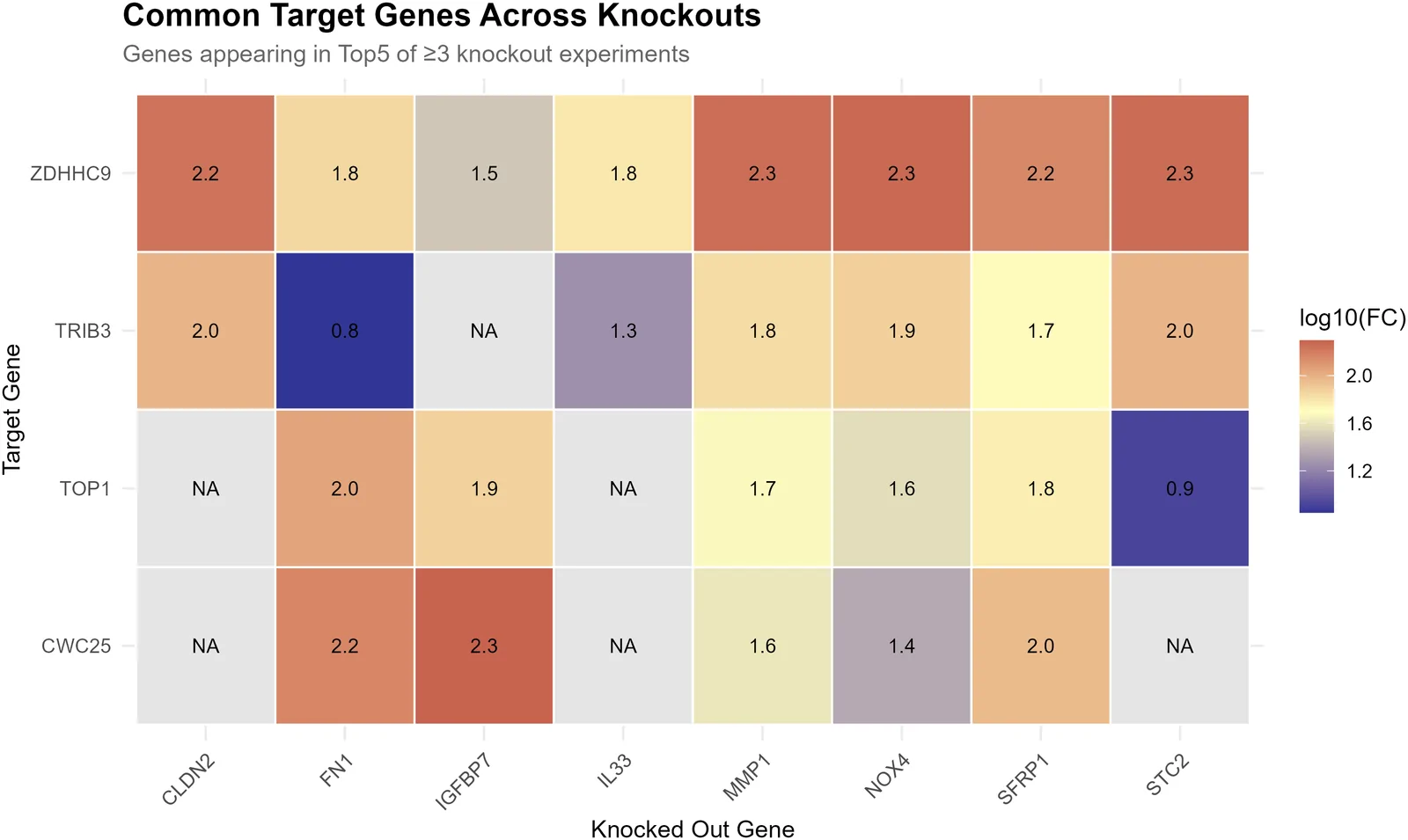
Heatmap of common target genes.

## Discussion

Cancer is typically driven by genetic and non-genetic alterations, and the identification of core genes is therefore of particular importance for targeted therapeutic strategies in specific malignancies. In this study, we propose a novel model designated as ChebTs, which integrates local topological features with a global attention mechanism, to explore biomarkers associated with the colorectal “adenoma-cancer” transition. Using ChebTs, we screened for key genes across multiple cancer types, and the results demonstrated that the incorporation of enhanced gene features effectively improved predictive performance, with the proposed model outperforming all baseline methods.

In this study, two datasets from the same microarray platform, GSE31905 and GSE41657, were obtained from the GEO database and subsequently merged. By integrating network topological features, differential gene expression profiles, and feature encodings, the model learned the connectivity relationships among genes and generated node representations that capture both neighboring node information and encoded features for model inputs. Interpretable analysis of the key genes was performed using GNNExplainer, which validated the reliability of the model [57]. At the same time, it is also acknowledged that GNNExplainer may have the limitation of not being able to fully capture the overall characteristics. To evaluate the robustness of the gene ranking in this study for the system, we conducted more than 5 independent repeated experiments. Under the condition that other conditions remained unchanged, different random seeds were used each time. We noticed that genes with lower importance have less significant contributions to the model prediction, and their rankings are more susceptible to random fluctuations. Therefore, the candidate gene selection in this study focuses on the stable core genes ranked in the top 10. The results suggested that eight genes, *FN1*, *CLDN2*, *IL-33*, *MMP1*, *STC2*, *IGFBP7*, *NOX4*, and *SFRP1* may serve as potential key biomarkers of the colorectal “adenoma-cancer” transformation process. Based on the PPI network from the STRING database, we found that *FN1* is the node with the highest connectivity among the eight genes, and it exhibits high-confidence interactions (score > 0.4) with multiple proteins. Among these, *FN1* is directly connected to *IGFBP7* (score = 0.432) and *NOX4* (score = 0.540), suggesting that *IGFBP7*, as an IGF signaling regulator, bridges the ECM adhesion signal with the growth factor signaling pathway. *NOX4*, a primary source of reactive oxygen species (ROS), its association with *FN1* implies that redox signaling may regulate cell migration and survival through the ECM microenvironment. Furthermore, *FN1* and *MMP1* are proposed to exert an indirect synergistic regulatory function via ECM proteases and inflammatory signals. Survival analysis further indicated that these genes are involved in three interrelated aspects, microenvironmental remodeling, metabolic reprogramming, and intrinsic cellular regulation. The eight key genes identified in this study do not function in isolation, rather, they constitute a collaborative gene regulatory network. These biological processes exhibit a structural feature of “partial overlap within modules and complementary functions between modules,” accurately reflecting the complexity of this pathological process, suggesting that they may cooperatively drive malignant transformation from adenoma to cancer. In addition, virtual knockout techniques were introduced to simulate network perturbations, thereby revealing the paradigmatic shifts of key genes during carcinogenesis. This approach allows the study to move beyond correlational screening toward functional validation of the identified genes [17].

This study revealed two important findings. First, the adenoma to cancer transition is not driven by mutations in individual genes but rather by the synergistic dysregulation of multiple genes at the network level. Previous studies have shown that numerous mutated genes exist in colonic crypts that drive the transformation of normal cells into cancer cells, however, the majority of these mutations do not progress to malignancy [1]. This observation suggests that a process of regulatory network remodeling occurs during the transition from benign to malignant states, and the eight genes identified in this study represent core nodes within this process. Second, analysis of common target genes through virtual knockout experiments revealed that *ZDHHC9* and *TRIB3* were significantly upregulated following gene knockout, suggesting that they may function as core downstream effectors. Studies have shown that [58] *ZDHHC9* enhances the transcriptional activity of c-Myc through palmitoylation, and activates the cAMP signaling pathway by using the *KLF5* gene, thereby promoting the proliferation and migration of cancer cells. Additionally, *ZDHHC9* can also upregulate the expression of PD-L1, facilitating tumor immune evasion. Based on these findings, we speculate that when genes such as *MMP1* and *NOX4* are knocked out, tumor cells will upregulate *ZDHHC9* to activate multiple proliferation pathways in response to compensatory adaptation to genetic stress, but it cannot be proven that there is a direct correlation between it and the core genes.

The enrichment results revealed a molecular network in which ECM remodeling serves as the central axis, with inflammation and TGF-β signaling synergistically driving malignant transformation in colorectal cancer. As a core component of the tumor microenvironment, the dynamic remodeling of the ECM plays a critical role in tumor invasion and metastasis [59]. The significant enrichment of ECM related pathways in this study suggests that the adenoma to cancer malignant transformation may be accompanied by qualitative and quantitative changes in ECM components. Indeed, previous studies have demonstrated that the expression levels of collagen I and IV are significantly higher in colorectal cancer tissues than in normal tissues, and that their expression correlates positively with the CAF marker α-SMA [60]. The enrichment of the TGF-β signaling pathway provides further insight into the upstream regulatory mechanisms governing ECM remodeling, and it may also establish a self-reinforcing vicious cycle [59]. Furthermore, the enrichment of inflammation related pathways such as AGE-RAGE and IL-17 suggests that the chronic inflammatory microenvironment may synergistically promote ECM remodeling and EMT by inducing oxidative stress and the release of pro-inflammatory factors [61]. In summary, the synergistic interaction between ECM remodeling and inflammatory signaling may be a key driver of malignant progression in colorectal cancer. Li et al. [62] discovered that bioactive compounds from food sources can regulate the molecular mechanisms of disease-related targets. This theory is highly consistent with the key gene characteristics screened in this study. Polyphenols, dietary fibers, and ω-3 fatty acids may simultaneously target multiple members of this feature and exert a synergistic preventive effect at different stages of cancer development. This understanding provides a theoretical foundation for the prevention strategy of colon cancer.

In recent years, deep learning has been widely applied to the prediction of cancer driver genes. For instance, Zamanitajeddin et al. [63] combined cellular networks with deep neural networks to achieve high accuracy prediction of gene mutation status. Wang et al. [29] employed an encoder based on energy constrained diffusion and attention mechanisms to effectively capture complex gene dependencies without being constrained by explicit gene-gene network relationships, enabling not only the identification of known oncogenes but also the discovery of previously unrecognized potential driver genes. However, existing methods still focus on the discrimination of static mutation states, primarily comparing differences between cancerous tissues and normal cells, making it difficult to capture network changes during dynamic processes [61]. In contrast, the present study emphasizes the adenoma to cancer dynamic transition, aiming to identify molecular biomarkers with high malignant potential and thereby facilitate precision intervention. Nevertheless, several limitations of this study should be acknowledged. First, the relatively small sample size may introduce a certain degree of bias, and future work will incorporate larger cohorts for validation. Second, although the results are supported by literature based validation and virtual knockout techniques, the lack of more robust in vitro experimental evidence remains a limitation. The virtual knockout technique, by simulating the impact of the target gene being knocked out on the network, cannot reveal the dynamic biological processes such as modification compensation and metabolic reprogramming that may occur after the actual gene knockout. Moreover, the prediction results of this technique may also deviate due to the quality of network construction and the selection of calculation parameters.

To provide more reliable biological validation of the genetic screening results in this study, we plan to use the zebrafish DSS + AOM chemical induction method to validate the screening results in future work. Based on GNNExplainer, we plan to validate the first three genes *FN1*, *CLDN2* and *IL-33*. First, adult zebrafish aged 3–4 months were selected to construct zebrafish strain *FN1*-KO using CRISPR. The reagent *FN1* Morpholino and CRISPR-Cas9 were prepared for gene knockdown. AOM pretreatment and DSS injection were used to induce colon adenoma formation. Seven control groups (n≥30 in each group) were set up and observed for 16 weeks. The evaluation indicators included histopathology (hematoxylin staining for counting adenomas and Alcian Blue staining for evaluating mucus barrier), molecular level detection (qPCR) and immunohistochemical verification (protein localization of *FN1*). Based on the above validation results, we plan to validate the protocol simultaneously for *CLDN2* and *IL-33*. This in vivo validation protocol will provide a key basis for elucidating the function of these genes in adenoma to cancer transformation of the colon. In future work, we will focus on extending the applicability of the proposed model beyond cancer gene screening to include the prediction of other complex diseases.
